# Circadian Lighting Was Associated with a Reduction in the Number of Hospitalized Patients Experiencing Falls: A Retrospective Observational Study

**DOI:** 10.3390/healthcare13141692

**Published:** 2025-07-14

**Authors:** Takeshi Okinami, Toshihiro Suzuki, Nobuyuki Nishikawa, Hiromitsu Negoro

**Affiliations:** 1 Department of Urology, Akashi Ninjyu Hospital, Akashi 674-0074, Japan; nnishikaw@gmail.com; 2 Yamada Shadowless Lamp Co., Ltd., Chiyoda-ku 101-0065, Japan; t_suzuki@skylux.co.jp; 3 Department of Urology, Institute of Medicine, University of Tsukuba, Tsukuba 305-8575, Japan; hnegoro@md.tsukuba.ac.jp

**Keywords:** circadian rhythm, melanopic light, fall prevention, aging, anticonvulsants

## Abstract

**Background**: Falls in hospitalized patients are a significant healthcare concern. This study examined whether circadian lighting, which helps to regulate circadian rhythms, reduces fall risk. **Methods**: A retrospective study was conducted in a 49-bed subacute and rehabilitation ward after the renovation and the installation of circadian lighting. Patients admitted during the five months with circadian lighting (intervention group) were compared to those admitted in the previous five months under fluorescent lighting (control group). Circadian lighting was defined as at least 275 equivalent melanopic lux between 7 a.m. and 12 p.m. **Results**: Significantly fewer patients in the intervention group experienced falls (7.4% vs. 15.0%, *p* = 0.0182). Logistic regression analysis identified circadian lighting as a protective factor (adjusted odds ratio [aOR] = 0.558, 95% confidence interval [CI]: 0.351–0.887, *p* = 0.0137), while age ≥ 80 (aOR = 2.48, 95% CI: 1.18–5.21, *p* = 0.0167) and anticonvulsant use (aOR = 3.68, 95% CI: 1.39–9.72, *p* = 0.0087) were significant risk factors. **Conclusion**: Circadian lighting was associated with a reduction in the number of patients who experienced falls, while advanced age and anticonvulsant use were significant risk factors.

## 1. Introduction

With the rapid aging of the population in recent years, the demand for the hospitalization of older patients has increased. Falls among hospital inpatients are the most frequently reported safety incident [1]. Reported rates of falls in hospital settings range from 5.71 to 18.0 per 1000 bed-days, with a trend toward higher rates in care wards for older adults [2]. The incidence of falls resulting in injury ranges from 30% to 51%, and the proportion of falls resulting in any fracture ranges from 1% to 3% [3]. Even falls that do not result in injury can trigger a fear of falling, anxiety, distress, depression, and reduced physical activity [4]. Furthermore, falls can lead to prolonged hospitalization and increased financial burden [5]. Dykes et al. reported that the average cost of a single fall was US$62,521 and that this cost did not differ significantly between injurious and non-injurious falls [6]. This represents a substantial economic burden on the healthcare system, as significant costs are incurred even in the absence of injury. Therefore, preventing falls in hospitalized patients is paramount.

Risk factors for patient-induced falls include recent falls, muscle weakness, behavioral disturbances, agitation, confusion, urinary incontinence or frequency, the use of “culprit” drugs, postural hypotension or syncope, advanced age, male sex, dementia, and delirium [3]. A systematic review of clinical practice guidelines by Montero-Odasso et al. highlights key recommendations for fall prevention that have garnered a strong consensus [7]. Among the most highly endorsed strategies are the stratification of fall risk (screening), assessment of gait and balance, implementation of multifactorial interventions (which concurrently address multiple risk factors), and a review of medications potentially increasing the risk of falls. In contrast, a systematic review and meta-analysis by Morris found that education was the only intervention associated with a statistically significant reduction in falls. Education for both patients and staff significantly decreased the rate of falls by 30% and the odds of a patient becoming a faller by 38% [8]. Nevertheless, a study by Heng et al. reported that the efficacy of educational interventions for fall prevention was diminished in patients with cognitive impairment [9]. This indicates a significant gap in effective, evidence-based fall prevention for patients with cognitive impairment, underscoring a critical need for the development of novel strategies.

We focused on delirium, urinary frequency, and dementia because these conditions have been associated with circadian rhythm disorders [10,11,12]. We postulated that circadian rhythm disruption contributes to an increased risk of falls by inducing delirium, frequent urination, and cognitive decline. Accordingly, this study aimed to determine if circadian lighting, by stabilizing the circadian rhythm, could mitigate these risk factors and consequently decrease the incidence of falls.

The circadian rhythm is one of the mechanisms that maintains homeostasis in the body, and light is a central modulator of this process [13]. Light influences this pathway when detected by intrinsically photosensitive retinal ganglion cells (ipRGCs) that send light intensity and wavelength information to the suprachiasmatic nucleus—the central clock—via the retinohypothalamic tract. Through this pathway, information about light reaches the pineal gland, where melatonin metabolism is controlled. Melatonin is known to promote sleep [14]. Melanopsin, the photopigment of ipRGCs, exhibits a peak spectral sensitivity to blue light at approximately 480 nm. The 480 nm nomogram was proposed as a “melanopic” photometric measurement related to melanopsin photoreception [15]. Using this this concept, the International WELL Building Institute adopted the “melanopic illuminance” approach that specifically incorporates the equivalent melanopic lux (EML), which reflects the contribution of ipRGCs [16]. The WELL guidelines recommend at least 275 EML light that starts no later than noon and lasts for at least 4 h. The renovation of our hospital ward presented a unique opportunity to install circadian lighting compliant with the WELL guidelines. We therefore designed this study to validate the hypothesis that such an intervention could decrease fall incidents among hospitalized patients.

## 2. Materials and Methods

### 2.1. Study Design

Our hospital has a total of 149 beds and provides care for patients who require subacute care, rehabilitation, and chronic care in a regional city in Japan. Two wards with subacute care and rehabilitation functions (a total of 49 beds) were installed with circadian lighting as part of a renovation and relocation project on 9 January 2024. The role of the two wards is to facilitate rehabilitation during the subacute phase of treatment. The intervention group included hospitalized patients in a post-renovation environment that included circadian lighting, whereas the historical control group included those in a pre-renovation environment with conventional fluorescent lighting. A retrospective observational study was conducted to compare the incidence of falls in both groups and identify the risk factors associated with fallers, who were defined as patients who experienced one or more falls. This study was approved by the Ethics Committee of Akashi Ninjyu Hospital (Akashi, Hyogo, Japan) under approval number 2024-1-002. This study was conducted in accordance with the Declaration of Helsinki. Information regarding the study was disseminated via hospital bulletin boards and the hospital website. Patients were provided with the opportunity to opt out of participation. Only data from patients who did not exercise this opt-out option were included in the analysis. The Ethics Committee of Akashi Ninjyu Hospital waived the requirement for individual informed consent. The authors accessed relevant data via the electronic medical record system between 1 November 2024 and 31 December 2024. Although the authors had access to identifiable patient records during the data collection phase, all data were fully anonymized prior to analysis.

### 2.2. Participants

**Figure 1:**
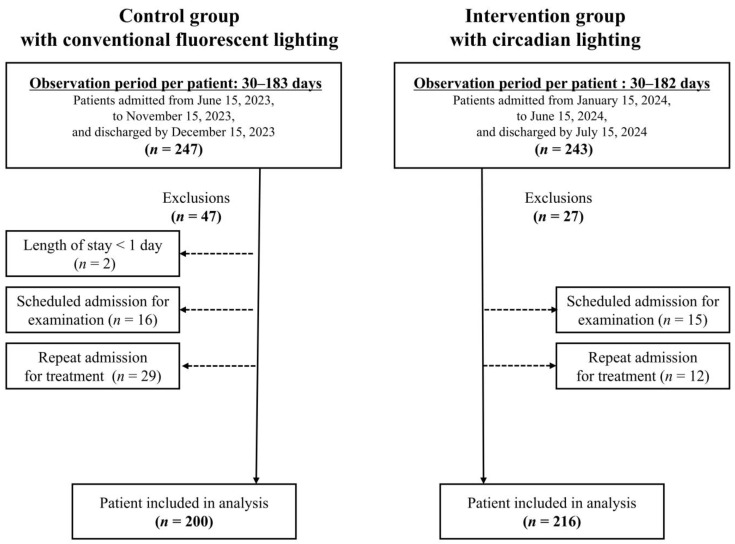
Flowchart of patient selection.

The intervention group consisted of patients who were admitted between 15 January 2024 and 15 June 2024 and subsequently discharged by 15 July 2024. The control group consisted of patients who were admitted between 15 June 2023 and 15 November 2023 and subsequently discharged by 15 December 2023. The study excluded patients who were admitted for less than one day or who were scheduled for 2 or 3 days of hospitalization for examination or treatment (Figure 1).

### 2.3. Lighting Setting

**Figure 2:**
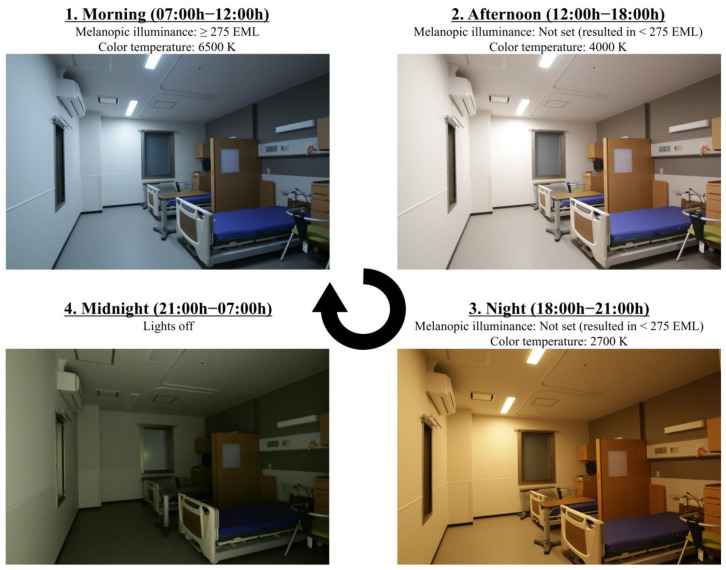
Circadian lighting in patient room.

The control group comprised 49 beds distributed across the second and third floors. Of these, 28 beds were located in fourteen south-facing rooms, 18 beds in four north-facing rooms, two beds in a west-facing room, and one bed in an east-facing room. The intervention group comprised 49 beds on the first floor that were distributed as follows: 21 beds in eight east-facing rooms, 11 beds in four south-facing rooms, and 17 beds in five west-facing rooms. The lighting in the control group was conventional fluorescent lighting, which predominantly consisted of 32 W and 40 W cyclic fluorescent lamps per bed. Patients and staff members were permitted to switch the lights on and off at their discretion; however, the lighting was typically switched on between 06:00 h and 07:00 h and off between 20:00 h and 21:00 h. The intervention group was provided with circadian lighting manufactured by Yamada Shadowless Lamp Corporation (Tokyo, Japan) that was designed in accordance with WELL guidelines. The lighting system includes a combination of two distinct types of light-emitting diodes (LEDs) and a control base. The LEDs (Dynasolis™; Nichia, Anan, Japan) are color adjustable by combining two types of LEDs: a high color temperature (azure color) with a higher output in the 480 nm spectrum and an incandescent lamp color (2700 K). The LED’s output was regulated with a control device (Kanda Tsushinki, Tokyo, Japan) to modify the illuminance and tone. In accordance with the WELL criteria, a light environment of at least 275 EML and a color temperature of 6500 K were implemented from 07:00 h to 12:00 h; the target melanopic illuminance was not established for the remaining periods. However, the color temperature was adjusted to 4000 K from 12:00 h to 18:00 h and to 2700 K from 18:00 h to 21:00 h, resulting in a reduced melanopic illuminance of less than 275 EML for each period. The lighting was scheduled to be turned off between the hours of 21:00 h and 08:00 h (Figure 2). The lighting system operated automatically and could not be manually controlled or overridden by patients. To prevent excessive evening melanopic illuminance in the intervention group, light-shielding curtains were employed in rooms where direct evening sunlight was strong (depending on season and window orientation) to minimize its ingress.

To evaluate the lighting in the control group, five south-facing rooms and two north-facing rooms were examined. The lighting assessments for the intervention group were examined for all 17 of the rooms. In the control group, the results were grouped and aggregated by the direction of window openings in the rooms to account for the influence of ambient light. In the intervention group, these rooms were considered as a single group for analysis purposes because most of the rooms had light-shielding curtains. The illuminance, color temperature, and melanopic illuminance in the area proximate to the pillows were recorded for each group at each time of day for two patterns: less ambient light exposure and actual ambient light exposure for actual use. The color temperature of the control group under actual operating conditions could not be measured near the pillows given the use of curtains around the bed; therefore, measurements in the center of the room were substituted. To evaluate the lighting environment in the absence of ambient light, the illuminance near the pillow was measured in the absence of the patient with light-shielding curtains on the windows and the lights on. Measurements for the conventional lighting environment were performed between 3 and 5 p.m., a timeframe deemed representative due to the minimal diurnal variation expected under conditions of reduced external light. The circadian lighting was measured for each of the three setting conditions: morning (07:00 h–12:00 h), afternoon (12:00 h–18:00 h), and night (18:00 h–21:00 h). The light environment was measured using a spectrometer (ISM-Lux; ISUZU OPTICS, Hsinchu, Taiwan). Subsequently, to assess the impact of ambient light, the lighting environment was evaluated under the actual conditions in which the patients were situated within the room. The illuminance near the pillow of each bed was measured without any restrictions on the opening and closing of the curtains. The conventional lighting was measured on two setting conditions: morning (09:00 h) and afternoon (13:00 h). In contrast, the LED lighting was measured on three setting conditions: morning (10:00 h), afternoon (15:00 h), and night (19:00 h). Because the aforementioned method was unsuitable given installation space limitations, an alternative method was employed for this purpose. An omnidirectional camera (THETA SC2; RICOH, Tokyo, Japan) was positioned at a height of 60 cm from the floor in the center of the room (at the height of the pillow) to capture images in all directions. The image data were then analyzed and measured using software (RELAPS, Omni Ver. 1.17.2202; Visual Technology Laboratory, Tokyo, Japan) to determine the illuminance of the pillow area. The light environments for conventional lighting and circadian lighting were measured on sunny days in 2023/10 and 2024/5, respectively. Melanopic illuminance was calculated in accordance with the methodology proposed by Lucas and colleagues [17,18].

### 2.4. Light Environment

In the control group, the melanopic illuminance in the south-facing rooms reached a sufficient level (526.3 EML) during the morning hours under actual operational conditions. However, in the north-facing rooms, the melanopic illuminance was insufficient at 60.4 EML (Table 1). In an environment with less ambient light, the desired melanopic illuminance could not be achieved. Regardless of the direction of the window opening, the melanopic illuminance was as low as 57.7 EML for south-facing rooms and 51.4 EML for north-facing rooms. In conclusion, the control group demonstrated the capacity to achieve sufficient melanopic illuminance in the presence of ambient light. However, when the influence of ambient light was reduced, such as in north-facing rooms or when shading curtains were utilized, the group exhibited an inability to attain sufficient melanopic illuminance. In contrast, the circadian lighting group was able to achieve the requisite melanopic illuminance of 315.7 EML in the morning, despite the influence of ambient light being blocked. Furthermore, the group was able to achieve 383.4 EML in actual operational conditions. In conclusion, the intervention group was able to provide sufficient melanopic illuminance in the morning, both in an environment blocked from the influence of ambient light and in actual operational conditions.

### 2.5. Variables

Patient information was collected retrospectively from the electronic medical record; age at admission, sex, body mass index (BMI), and the primary reason for admission (classified into four categories: orthopedic and dermatological diseases, internal medicine diseases, cerebrovascular and neurological diseases, and malignant tumors) were recorded. Medical history was recorded as the presence or absence of five diseases (cerebrovascular disease, Parkinson’s disease, other neurological diseases, diabetes mellitus, and active malignancy). The motor items score on the Functional Independence Measure (FIM) [19] were used to evaluate activities of daily living (ADLs), whereas the Mini-Mental State Examination (MMSE) scores [20] were employed to assess cognitive function. Additionally, the length of hospitalization, outcome, and medications administered within the initial seven-day period were evaluated. The data regarding medications were classified and tabulated by referencing existing reports [21,22]. Sleep medications were subdivided into two groups: benzodiazepines (BZDs)/Z-drugs and other hypnotics that included melatonin receptor agonists and orexin receptor antagonists.

### 2.6. Study Procedure

A fall was defined as “an event which results in a person coming to rest inadvertently on the ground or floor or other lower level” as defined by the World Health Organization [23]. To determine the incidence of falls, a search was conducted using the keywords “fall” and “discovery” in the participants’ medical records. To ensure data completeness and minimize the likelihood of underreporting, fall records were sourced from routine daily nursing documentation instead of relying solely on an incident reporting system. Furthermore, data on the date, time, circumstances, and occurrence of adverse events for each fall were collected and analyzed by referencing existing reports [24]. Patients who experienced one or more falls were classified as “fallers,” and the number of fallers was investigated. The number of fall incidents was adjusted to reflect the number per 1000 patient-days to facilitate comparison with findings from prior epidemiological studies.

### 2.7. Statistical Analysis

The backgrounds of patients in the two groups were statistically compared using Fisher’s exact test and the Mann–Whitney U test. The number of fallers was compared between the two groups using Fisher’s exact test. Additionally, the fallers’ backgrounds and the summary of falls in the control and intervention groups were compared using Fisher’s exact test and the Mann–Whitney U test. To investigate the factors associated with falls, the patients in the control and intervention groups were aggregated and divided into two groups: fallers and non-fallers. The differences between the two groups were then compared. For each numerical data set, the patients were divided into two groups using the following cutoff values: 80 years of age or older, a BMI of 18.5 or higher, a score of 27 or higher for FIM motor items, and a score of 24 or higher on the MMSE. The differences between the two groups for each item were compared using Fisher’s exact test. Additionally, a logistic regression analysis was performed to evaluate the risk factors for fallers. To select independent variables for the final multivariate logistic regression model, we initially utilized a LASSO (least absolute shrinkage and selection operator) regression analysis. This approach was chosen to objectively identify the most important predictors from a pool of 25 candidate variables while mitigating the risk of overfitting. A LASSO logistic regression model was fitted with fall occurrence (yes/no) as the dependent variable. The optimal L1 penalty tuning parameter (λ) was identified via 10-fold cross-validation, applying the one-standard-error (1-SE) rule. We then sought to advance all variables with non-zero coefficients from the selected LASSO model to the multivariate analysis. However, this procedure resulted in no variables being retained. We selected five independent variables for the multivariate analysis. Alongside our primary variable, the circadian lighting environment, four other variables were selected based on findings from previous studies. This yielded an events per variable (EPV) of 9.2 based on 46 fall events. The model then underwent rigorous diagnostic testing. First, to assess for multicollinearity, variance inflation factors (VIFs) were calculated for all variables; none exceeded the pre-specified threshold of 10. Next, the model’s overall goodness-of-fit was evaluated using the Hosmer–Lemeshow test. Finally, Cook’s distance was analyzed to ensure that no single observation exerted undue influence on the model. Statistical analyses in this study were conducted using EZR (Version 1.6.8) [25]. The data set included missing values for several variables, including BMI, the FIM motor items score, and the MMSE score. To address this issue, the available case analysis approach, which entails evaluating all available cases separately for each examined variable, was used. The level of statistical significance was set at *p* < 0.05.

## 3. Results

### 3.1. Participant Demographics

The control group consisted of 200 patients and the intervention group comprised 216 patients. The study was conducted in Japan, and all recruited participants were of Japanese ethnicity. The study population was predominantly elderly, characterized by a mean age of 82 years, with 61.5% of individuals aged 80 or older. The median score for FIM motor items was 21.0, indicating that a significant proportion of patients required a moderate level of assistance. Similarly, the median MMSE score was 21.0, indicating that a substantial proportion of participants experienced cognitive decline. Among the patients with primary illnesses, 53.6% had internal medicine diseases, 24.5% had a history of stroke, and 12.5% had a history of Parkinson’s disease. The median length of stay was 18.0 days. Patient outcomes were stratified into three categories. The majority (56.5%) were discharged from the ward, while 29.1% remained for continued care. The third group (14.4%) comprised patients who were transferred due to a worsening clinical status or who died. For this cohort, the analysis was censored at the date of the event. Collectively, the study population represented a diverse cohort of patients. No significant differences in patient background characteristics were observed between the two groups for any of the measured items (Table 2).

### 3.2. Comparison of the Number of Fallers and Falls

In the control group, 30 patients experienced at least one fall compared with 16 patients in the intervention group (15.0% vs. 7.4%, *p* = 0.0182; Table 3). The number of falls per 1000 patient-days was 7.14 in the control group and 4.43 in the intervention group (rate ratio = 0.62). Severe adverse events associated with falls were noted only in the control group (three cases: one sciatic fracture, one rib fracture, and one subdural hematoma with rib fracture). The incidence of falls involving serious complications was 0.55 falls per 1000 patient-days in the control group and 0 per 1000 patient-days in the intervention group. Minor adverse events (such as pain and swelling) that were related to falls were recorded in 13 patients in the control group and 4 patients in the intervention group (Table S1). No significant differences were observed in the backgrounds of patients who fell (Table S2) or in the characteristics of fall incidents (Table S1).

### 3.3. Risk Factors for Fallers

To investigate risk factors for falls, patients in the control and intervention groups were pooled and then subdivided into “fallers” and “non-fallers.” To profile the patients who experienced falls, baseline characteristics were compared between the faller and non-faller groups via univariate analysis using Fisher’s exact test. Univariate analysis showed that fallers were significantly more likely to be over 80 years of age and taking anticonvulsant medications. Conversely, fallers were significantly less likely to receive circadian lighting (Table 4). We selected five variables for the multivariate analysis. In addition to the circadian lighting environment, we included four variables identified as risk factors in prior research [21,22,26,27,28]: older age (≥80 years), medications for dementia, use of BZD/Z-drugs, and use of anticonvulsants. Preliminary analyses confirmed the model’s suitability for multivariate logistic regression. We found no evidence of multicollinearity (all VIFs < 1.05). Model diagnostics were also satisfactory, with the Hosmer–Lemeshow test indicating a good model fit (χ^2^ = 1.0629, df = 4, *p* = 0.9001) and no influential outliers identified via Cook’s distance. Accordingly, we conducted a multivariate logistic regression analysis to identify risk factors for patients who experienced falls. After adjusting for these confounders, circadian lighting, older age (≥80 years), and the use of anticonvulsants were significant factors for patients who experienced falls, with adjusted odds ratios of 0.558 (95% confidence interval [CI]: 0.351–0.887, *p* = 0.0137), 2.48 (95% CI: 1.18–5.21, *p* = 0.0167), and 3.68 (95% CI: 1.39–9.72, *p* = 0.0087), respectively (Table 5).

## 4. Discussion

### 4.1. Study Strength

This study examined the effects of circadian lighting in a hospital setting. The findings indicated that the implementation of circadian lighting, which supports the regulation of circadian rhythms, was significantly associated with a reduction in the number of fallers.

### 4.2. Lighting Environment

The circadian lighting systems modulate melanopic illuminance through the control of color temperature. This capability enables the provision of sufficient melanopic illuminance during morning hours, even under conditions of low ambient light, while concurrently reducing melanopic illuminance in the evening and at night prior to sleep onset. Such dynamic regulation is unachievable with conventional lighting systems, as they typically deliver a constant level of melanopic illuminance. Circadian lighting consistently adjusts and maintains the appropriate melanopic illuminance levels, synchronized with the diurnal cycle, irrespective of external variables such as window orientation, prevailing weather conditions, or the presence of shading elements. Integration with blackout curtains further serves to curtail the ingress of external evening light, thereby preventing excessive melanopic illuminance. This approach can mitigate phase deviations in the circadian clock by ensuring sufficient exposure to morning melanopic illuminance while restricting excessive melanopic illuminance during evening hours (Table 1).

### 4.3. Mechanisms Underlying the Reduction in the Number of Fallers

The precise mechanism by which circadian lighting is associated with a reduction in the number of fallers remains unclear. No significant differences in patient background or the specific circumstances of falls were observed between the two groups (Tables S1 and S2). Therefore, circadian lighting might not reduce falls related to any single cause or circumstance. Rubenstein’s review [29] established that most falls are associated with identifiable risk factors (e.g., weakness, unsteady gait, confusion, and psychoactive medications), indicating that falls may be influenced not only by physical function but also by psychological function. In a previous study, Hitcho and colleagues reported that approximately 50% of falls were associated with the act of defecation [24]. Similarly, 17 of the 32 identified causes of falls (53%) in this study were related to defecation. Of the 33 nighttime falls (9 p.m.–7 a.m.) that occurred across both groups, 10 of the 14 cases (71.4%) for which a behavioral trigger could be identified were associated with toileting-related activities. Consistent with previous reports, this suggests that nocturia can be a significant precipitating factor for falls. In the context of nocturia, both increased nocturnal urine volume and decreased nocturnal functional bladder capacity have been reported to be associated with circadian rhythms [30]. Alzheimer’s disease has also been associated with disrupted circadian rhythm [12,31]. Thus, by contributing to improving urinary frequency and psychological function, circadian lighting may reduce the incidence of fallers. An elevated risk of falls has been associated with the use of BZDs [28]. Moreover, zolpidem—a Z-drug—was notably correlated with an increased risk of falls (adjusted odds ratio 4.37, 95% CI = 3.34–5.76, *p* < 0.001) [32]. However, BZDs/Z-drug medications were not identified as risk factors for fallers in this study.

### 4.4. Fall Prevention

The number of falls in the control group was consistent with that in previous reports. A key finding was the remarkably low incidence of falls in the intervention group (4.43 per 1000 patient-days). This outcome is especially significant in the context of our high-risk population, which was characterized by its advanced age (61.5% aged ≥80 years) and a high prevalence of cognitive impairment—a condition known to limit the effectiveness of traditional educational interventions. Miake-Lye et al. reported that high-quality, multifactorial interventions reduce falls in hospitals by up to 30% [4]. Similarly, Morris et al. observed that up to 30% of falls can be prevented by assessing risks and taking appropriate measures [1]. A review by Cameron et al. suggested that the evidence supporting the effectiveness of fall-risk reduction strategies in hospitals remains inconclusive [2]. Although multifactorial interventions have reduced fall rates (relative risk 0.80), their benefits may be most applicable in subacute settings. Karlsson’s review showed that exercise can lower both the number of fallers and the overall fall rate (rate ratio = 0.63–0.78) in the local population, but its efficacy in care facilities and hospitals remains inconclusive [33]. This discrepancy represents a considerable challenge for researchers and healthcare professionals in identifying effective fall prevention strategies in hospitalized patients. This study showed that circadian lighting was associated with a 50.7% reduction in the number of fallers. The incidence of falls decreased (rate ratio = 0.62). Haines et al. reported that a comprehensive patient education program reduced falls by 26%, whereas educational materials alone resulted in a 16% reduction. These effects were not observed in patients with cognitive impairment [34]. Therefore, such programs are expected to be less effective in older populations with cognitive decline. Nevertheless, the study’s intervention group revealed preventive benefits, suggesting that circadian lighting may be particularly useful for older adults with cognitive impairment. Combining circadian lighting with education on fall prevention could potentially enhance this protective effect. Notably, falls with serious complications were not observed in the intervention group. This outcome is unlikely solely attributable to circadian lighting. Floor structure may have been a contributing factor given that the floors in the control group were concrete with vinyl tiles, whereas those in the intervention group were floating floors supported by baseboards and support legs that mitigate impact forces in the event of falls.

### 4.5. Future Prospects

Regulating circadian rhythms through lighting closely mirrors physiological processes and is unlikely to produce adverse events. If circadian lighting effectively reduces the number of fallers among hospitalized patients, it has the potential to lower both the physical and psychological burdens on patients, decrease the workload for healthcare personnel, and reduce medical costs by preventing complications. These outcomes would significantly benefit both patients and staff. One limitation of circadian lighting is the high cost of the required equipment, which may be a barrier to widespread adoption. However, our findings indicated that patients can achieve adequate melanopic illuminance in a conventional lighting environment if sufficient ambient light is available. For patients who lack adequate morning melanopic exposure, spending time in a well-lit area could provide an effect similar to that of circadian lighting. This approach offers a more affordable alternative when full-scale implementation of a circadian lighting system is not feasible. Establishing a definitive link between circadian rhythms and falls would also open up the possibility of prospectively evaluating the fall prevention potential of pharmacological agents that leverage these mechanisms, such as the melatonin receptor agonists and orexin receptor antagonists already used to treat insomnia. An integrative review by Pati et al. on architectural factors for fall prevention identified numerous elements, like the layout of patient rooms, bathrooms, doors, beds, flooring, chairs, and lighting [35]. However, the review also highlighted that robust scientific evidence supporting these interventions remains limited. In this context, circadian lighting could represent a novel, evidence-based architectural approach to reducing falls.

### 4.6. Limitations

This study has several limitations. Our central hypothesis was that the disruption of patients’ circadian rhythms could lead to delirium, frequent urination, and cognitive decline, consequently increasing the incidence of falls. We anticipated that the introduction of circadian lighting would help to normalize these rhythms, thereby mitigating the aforementioned symptoms and ultimately reducing fall incidents. A primary limitation is that we did not directly assess the impact of circadian lighting on the patients’ circadian rhythms. Useful, minimally invasive, and quantitative methods for this purpose include the measurement of salivary melatonin, urinary 6-sulfatoxymelatonin (a major melatonin metabolite) [36], and core body temperature rhythms using wearable devices [37]. Future research employing circadian lighting should incorporate these direct measurements. Furthermore, we were unable to evaluate changes in nocturia, delirium, or cognitive function over time. For an objective assessment of nocturia, a bladder diary is a useful and minimally invasive tool for measuring nocturnal urine volume, voiding frequency, and bladder capacity. To rigorously examine the effect of circadian lighting on nocturia, it would be necessary to compare the same patients under both conventional and circadian lighting conditions; this remains a topic for future research. Likewise, the longitudinal assessment of delirium and cognitive function is also a task for subsequent studies. This study included a significant number of patients with cognitive decline, as indicated by low MMSE scores. While disruptions in circadian rhythms have been reported in patients with cognitive impairment, our study could not determine whether the efficacy of circadian lighting in preventing falls was specific to this population or if it would be effective in patients without cognitive decline as well. Next, it is important to acknowledge the potential for bias and confounding, as this was a retrospective, single-center study with non-randomized assignment to the control and intervention groups. Being a single-center study, there is a likely selection bias, given that our hospital specializes in post-acute care. To address this issue, a multicenter study involving hospitals with diverse functions would be necessary.

Additionally, it is plausible that unmeasured confounding factors influenced the fall risk. Specifically, the following potential confounders were not accounted for:

- Reliability of nursing records: As fall documentation depended on nursing records, it is possible that some falls were not recorded if the nursing staff was unaware of them.
- Adherence to the lighting environment: In the control group, patients could freely operate their room lights, whereas in the intervention group, the circadian lighting was generally not user-adjustable. However, patients in both groups could control their bedside lights, which may have influenced the outcome.
- Changes in hospital facilities from renovation: Environmental changes beyond lighting, resulting from the hospital renovation, are another source of confounding. The renovation involved changes to room entrances, toilet access, and corridors. However, due to building regulations, there were no major changes to the surface area of the rooms. Considering that the majority of falls (81.5%) occurred within patient rooms, we concluded that structural changes outside the rooms were unlikely to have had a significant impact on the fall incidence rate.
- Organizational factors: Because the two groups were studied during different periods, differences in the attending healthcare staff could be a confounding factor. As reported by Kim et al., the likelihood of patient falls is significantly lower in hospitals with lower nurse-to-patient ratios and longer care times, whereas the risk is markedly higher in hospitals with a greater proportion of novice nurses with less than one year of clinical experience [38]. This suggests that organizational-level factors may influence fall risk. However, the medical functions of the ward remained consistent throughout both study periods, the attending physicians did not change, and only a few nurses were different between the periods.
- Seasonal factors: The different seasons during which the two groups were studied could also act as a confounder. Fall events can fluctuate seasonally, and a prior study has reported fewer falls in autumn than in spring [39]. Given the composition of our study—the control group was hospitalized during a season with typically fewer falls, while the intervention group was hospitalized during a season with more falls—it is possible that an even more favorable fall prevention effect would have been observed in the intervention group if the seasonal factor had been controlled.

A statistical limitation of this study is that the EPV for the multivariate logistic regression model was 9.2, which is below the common threshold of 10. Consequently, the possibility of overfitting cannot be entirely ruled out. Therefore, the results of this study should be interpreted with caution, and validation with a larger sample size is warranted. To resolve these issues, a prospective, multicenter, randomized controlled trial is required. From a more experimental viewpoint, an ideal design would involve equipping the same patient rooms with both conventional and circadian lighting systems that can be switched. However, given that the fall incidence in our control group was not high (3.9 falls per 1000 patient-days), assessing the effect of switching lighting for individual patients would be difficult. It would therefore be necessary to evaluate the fall prevention effect in randomized patient groups over a sufficient observation period. To account for seasonal factors, the observation periods for the control and intervention groups should be set during the same season; an observation period of one year would be preferable to allow for comparison with other reports. Furthermore, a robust system for ensuring the reliable documentation of all fall events is essential.

However, a practical challenge for circadian lighting is the significant implementation cost, which may render large-scale studies difficult. A potential alternative may be to utilize natural daylight. If it can be demonstrated that sufficient exposure to morning sunlight provides an adequate amount of melanopic illuminance, and if the resulting control of circadian rhythms is verified with objective markers such as urinary melatonin metabolite levels, this approach could be considered to have an equivalent effect. This would enable more medical institutions to investigate the association between circadian rhythms and falls at a lower cost.

## 5. Conclusions

The introduction of circadian lighting was shown to reduce the number of inpatients experiencing falls within a population comprising predominantly elderly individuals (61.5% aged 80 or older) and including many patients with cognitive impairment. Although the direct mechanism remains unclear and the results must be interpreted with caution due to numerous limitations from the retrospective, non-randomized design, circadian lighting shows potential as a novel approach for fall prevention. Patient education, a primary and established prevention method, faces the challenge of limited efficacy in patients with cognitive impairment. Our results, however, suggest that circadian lighting may be effective for this very population. While it requires an initial installation cost, circadian lighting offers the advantage of not needing the ongoing investment of human resources.

Future multicenter, prospective, randomized controlled trials are strongly desired to further investigate the impact of circadian rhythms on inpatient falls in greater detail. Furthermore, there is potential to develop more accessible fall prevention strategies that operate through the circadian mechanism. Even without formal circadian lighting, interventions such as ensuring sufficient exposure to morning daylight or using pharmacological treatments to modulate circadian rhythms could offer promising, more easily implemented alternatives.

## Figures and Tables

**Table 1:** Lighting circumstance.

			Melanopic Illuminance (EML)		Color Temperature (K)		Illuminance (lx)		Number of Rooms
			Median	IQR	Median	IQR	Median	IQR	
**Reduced ambient light exposure (shade condition)**									
Control group	South-facing room	p.m.	57.7	[51.1–94.0]	3630	[3456–3715]	128.7	[113.7–159.2]	5
	North-facing room		51.4	[36.3–66.5]	3687	[3618–3755]	104.6	[74.8–134.4]	2
Intervention group		a.m.	315.7	[245.3–323.9]	6497	[6460–6511]	279.4	[217.1–286.6]	17
		p.m.	151.7	[132.7–207.3]	4348	[4121–4395]	189.7	[165.8–259.1]	17
		night	54.0	[49.5–68.2]	2627	[2615–2656]	120.0	[109.9–151.6]	17
**Actual ambient light exposure (normal use)**									
Control group	South-facing room	a.m.	526.3	[470.8–722.4]	5268 *	[5131–5674]	634.9	[585.4–882.2]	5
		p.m.	1794.9	[1056.2–1913.8]	4514 *	[4404–5432]	1929.3	[1236.7–2407.5]	5
	North-facing room	a.m.	60.4	[60.1–60.7]	4070 *	[3822–4317]	105.1	[95.3–114.9]	2
		p.m.	59.3	[58.0–60.6]	3901 *	[3693–4108]	95.8	[90.7–101.0]	2
Intervention group		a.m.	383.4	[245.9–565.7]	6015	[5548–6203]	372.0	[222.0–514.0]	17
		p.m.	271.5	[201.8–647.2]	4323	[4056–4702]	316.0	[253.0–649.0]	17
		night	39.9	[26.9–81.1]	2606	[2543–2634]	94.0	[70.0–183.0]	17

* The measurements were taken at the center of the room. IQR; interquartile range.

**Table 2:** Participant demographics.

		Control Group	Intervention Group	*p* Value
Factors	Number of Cases	200	216	
Age, median [range]		82.00 [37.00–97.00]	83.00 [35.00–99.00]	0.314
Sex, *n* (%)	Men	83 (41.5)	80 (37.0)	0.367
	Women	117 (58.5)	136 (63.0)	
Ethnicity, *n* (%)	Japanese	200 (100.0)	216 (100.0)	1
Body mass index (kg/m^2^), median [range] *		20.28 [11.60–35.28]	19.97 [11.89–38.14]	0.662
FIM motor items score, median [range] *		23.00 [13.00–72.00]	20.00 [13.00–77.00]	0.612
MMSE score, median [range] *		20.50 [4.00–30.00]	21.00 [1.00–30.00]	0.547
Primary illness, *n* (%)	Orthopedics or dermatology	55 (27.5)	66 (30.6)	0.142
	Various internal diseases **	110 (55.0)	113 (52.3)	
	Neurological disorder	26 (13.0)	18 (8.3)	
	Malignancy	9 (4.5)	19 (8.8)	
Past history, *n* (%)	Stroke	53 (26.5)	49 (22.7)	0.425
	Parkinson’s disease	27 (13.5)	25 (11.6)	0.557
	Other neurological disease	23 (11.5)	16 (7.4)	0.179
	Diabetes mellitus	55 (27.5)	55 (25.5)	0.658
	Malignancy	16 (8.0)	29 (13.4)	0.083
Medication within 7 days of hospitalization, *n* (%)	Diuretics	53 (26.5)	57 (26.4)	1
	Antihypertensives except diuretics	85 (42.5)	105 (48.6)	0.237
	Anti-coagulants	63 (31.5)	66 (30.6)	0.916
	Diabetes medication	39 (19.5)	38 (17.6)	0.705
	BZD/Z-drugs	36 (18.0)	35 (16.2)	0.696
	Other hypnotics ***	65 (32.5)	71 (32.9)	1
	Anti-depressants	17 (8.5)	21 (9.7)	0.735
	Anti-dementia medication	30 (15.0)	27 (12.5)	0.479
	Anticonvulsants	13 (6.5)	15 (6.9)	1
	Antipsychotics	44 (22.0)	56 (25.9)	0.361
	Antihistamines	18 (9.0)	14 (6.5)	0.362
	Non-narcotic analgesics	5 (2.5)	10 (4.6)	0.298
	Narcotic analgesics	7 (3.5)	8 (3.7)	1
Length of stay, days	median [IQR]	17.50 [9.00–36.25]	18.50 [8.00–39.25]	0.699
	mean (SD)	27.33 (24.40)	27.15 (24.83)	
Outcome	Discharge	107 (53.5)	128 (59.3)	0.241
	Transfer to a different ward	64 (32.0)	57 (26.4)	
	Transfer to a different hospital	19 (9.5)	14 (6.5)	
	Death	10 (5.0)	17 (7.9)	

* Excludes some missing data. There was missing data for BMI in 13 (control) and 17 (intervention) cases. Missing data for FIM motor items score was seen in 8 (control) and 13 (intervention) cases. For MMSE, there was missing data in 112 (control) and 103 (intervention) cases. ** Includes cardiovascular, gastroenterology, otolaryngology, respiratory, and urology. *** Includes melatonin receptor agonists and orexin receptor antagonists. FIM: Functional Independence Measure, MMSE: Mini-Mental State Examination, BZD: benzodiazepine, IQR: interquartile range, SD: standard deviation.

**Table 3:** Between-group comparisons of fall-related outcomes.

	Control Group	Intervention Group	
Patients, *n*	200	216	
Patient days	5465	5864	
Fallers, *n* (%)	30 (15.0%)	16 (7.4%)	*p* = 0.0182
Falls, *n* (per 1000 patient-days)	39 (7.14)	26 (4.43)	
Falls with serious complications, *n* (per 1000 patient-days)	3 (0.55) *	0 (0)	

* One ischial fracture, one rib fracture, and one subdural hematoma and rib fracture.

**Table 4:** Fallers’ risk factors (univariate analysis).

		Non-Faller	Faller	*p* Value
Factors	Number of Cases	370	46	
Age in years, *n* (%)	≤79	149 (40.3)	11 (23.9)	0.036
	≥80	221 (59.7)	35 (76.1)	
Sex, *n* (%)	Men	144 (38.9)	19 (41.3)	0.751
	Women	226 (61.1)	27 (58.7)	
Body mass index in kg/m^2^, *n* (%) *	<18.5 (underweight)	123 (35.8)	15 (35.7)	1
	≥18.5	221 (64.2)	27 (64.3)	
FIM motor items score (%) *	≤26 (poor motor ADL)	209 (59.5)	22 (50.0)	0.257
	≥27	142 (40.5)	22 (50.0)	
MMSE score (%) *	≤23 (cognitive decline)	109 (64.5)	25 (78.1)	0.156
	≥24	60 (35.5)	7 (21.9)	0.156
Lighting, *n* (%)	Conventional fluorescent light	170 (45.9)	30 (65.2)	0.018
	Circadian LED light	200 (54.1)	16 (34.8)	
Primary illness, *n* (%)	Orthopedics or dermatology	110 (29.7)	11 (23.9)	0.858
	Various internal diseases **	196 (53.0)	27 (58.7)	
	Neurological disorder	39 (10.5)	5 (10.9)	
	Malignancy	25 (6.8)	3 (6.5)	
Past history, *n* (%)	Stroke	91 (24.6)	11 (23.9)	1
	Parkinson’s disease	49 (13.2)	3 (6.5)	0.242
	Other neurological disease	33 (8.9)	6 (13.0)	0.417
	Diabetes mellitus	96 (25.9)	14 (30.4)	0.595
	Malignancy	41 (11.1)	4 (8.7)	0.803
Medication within 7 days of hospitalization, *n* (%)	Diuretics	95 (25.7)	15 (32.6)	0.375
	Antihypertensives except diuretics	174 (47.0)	16 (34.8)	0.142
	Anti-coagulants	117 (31.6)	12 (26.1)	0.502
	Diabetes medication	66 (17.8)	11 (23.9)	0.317
	BZD/Z-drugs	61 (16.5)	10 (21.7)	0.405
	Other hypnotics ***	118 (31.9)	18 (39.1)	0.322
	Anti-depressants	36 (9.7)	2 (4.3)	0.411
	Anti-dementia medication	46 (12.4)	11 (23.9)	0.041
	Anticonvulsants	21 (5.7)	7 (15.2)	0.025
	Antipsychotics	85 (23.0)	15 (32.6)	0.148
	Antihistamines	31 (8.4)	1 (2.2)	0.235
	Non-narcotic analgesics	13 (3.5)	2 (4.3)	0.676
	Narcotic analgesics	13 (3.5)	2 (4.3)	0.676
Length of stay, *n* (%)	≤29 days	252 (68.1)	25 (54.3)	0.069
	≥30 days	118 (31.9)	21 (45.7)	
Outcome, *n* (%)	Discharge	207 (55.9)	28 (60.9)	0.703
	Transfer to a different ward	107 (28.9)	14 (30.4)	
	Transfer to a different hospital	30 (8.1)	3 (6.5)	
	Death	26 (7.0)	1 (2.2)	

* Excludes some missing data. There was missing data for BMI in 26 (non-faller) and 4 (faller) cases. Missing data for FIM motor items score was seen in 19 (non-faller) and 2 (faller) cases. For MMSE, there was missing data in 201 (non-faller) and 14 (faller) cases. ** Includes cardiovascular, gastroenterology, otolaryngology, respiratory, and urology. *** Includes melatonin receptor agonists and orexin receptor antagonists. FIM: Functional Independence Measure, MMSE: Mini-Mental State Examination, BZD: benzodiazepine.

**Table 5:** Fallers’ risk factors (multivariable analysis).

	Faller		
Factors	Adjusted Odds Ratio	95% Confidence Interval	*p* Value
Circadian lighting	0.558	0.351–0.887	0.0137
Age ≥ 80 years	2.48	1.18–5.21	0.0167
Anticonvulsant medication	3.68	1.39–9.72	0.0087
Anti-dementia medication	2.07	0.954–4.48	0.0657
BZDs/Z-drugs	1.46	0.667–3.19	0.344

BZD; benzodiazepine.

## Data Availability

The data presented in this study are available on request from the corresponding author due to privacy or ethical reasons.

## Supplementary Materials

The following supporting information can be downloaded at: https://www.mdpi.com/article/10.3390/healthcare13141692/s1, Table S1: Patterns of falls between groups; Table S2: Faller demographics.

## Abbreviations

The following abbreviations are used in this manuscript:

aOR	adjusted odds ratio
CI	confidence interval
ipRGCs	intrinsically photosensitive retinal ganglion cells
EML	equivalent melanopic lux
LEDs	light-emitting diodes
BMI	body mass index
FIM	Functional Independence Measure
ADLs	activities of daily living
MMSE	Mini-Mental State Examination
BZDs	benzodiazepines
LASSO	least absolute shrinkage and selection operator)
EPV	events per variable
VIF	variance inflation factor
IQR	interquartile range
SD	standard deviation
