# Supplementary material for: Circadian Lighting Was Associated with a Reduction in the Number of Hospitalized Patients Experiencing Falls: A Retrospective Observational Study
Source: Healthcare (Basel). 2025 Jul 14;13(14):1692. doi: 10.3390/healthcare13141692 (PMC12294480; doi:10.3390/healthcare13141692)
Supplement: Supplementary file 1 [file healthcare-13-01692-s001.zip › healthcare-3676209-supplementary.pdf]

Circadian lighting was associated with a reduction in the number of hospitalized patients experiencing falls: A retrospective observational study

Supplementary Material

Table S1. Patterns of falls between groups
Table S2. Faller demographics

Table S1. Patterns of falls between groups

		Control group	Intervention group	p value
**Factor**	Number of cases	39	26	
**Onset-to-admission interval, median [range]**		7.0 [0–51]	8.5 [1–70]	0.212
**Location**	Patient room	30 (76.9)	23 (88.5)	0.389
	Toilet	2 (5.1)	0 (0.0)	
	Bathroom	0 (0.0)	1 (3.8)	
	Other	6 (15.4)	2 (7.7)	
	Unknown	1 (2.6)	0 (0.0)	
**Time of day**	7:00 a.m.–8:59 p.m.	18 (46.2)	14 (53.8)	0.617
	9:00 p.m.–6:59 a.m.	21 (53.8)	12 (46.2)	
**Discovery type**	Found on floor	30 (76.9)	16 (61.5)	0.379
	Witnessed	5 (12.8)	6 (23.1)	
	Self-reported	4 (10.3)	4 (15.4)	
**Assistance type at time of fall**	Unassisted	39 (100.0)	24 (92.3)	0.156
	Assisted by employee	0 (0.0)	2 (7.7)	
**Fall type**	Collapsed	9 (23.1)	8 (30.8)	0.808
	Slid to floor	7 (17.9)	3 (11.5)	
	Fell from height	1 (2.6)	0 (0.0)	
	Unknown	22 (56.4)	15 (57.7)	
**Fall mechanism**	Slipped or tripped	8 (20.5)	3 (11.5)	0.235
	Muscle weakness	4 (10.3)	2 (7.7)	
	Lost balance	3 (7.7)	7 (26.9)	
	Dizziness or fainting	2 (5.1)	0 (0.0)	
	Unknown	22 (56.4)	14 (53.8)	
**Activity at time of fall**	Getting out of bed	10 (25.6)	2 (7.7)	0.192
	Ambulating	4 (10.3)	4 (15.4)	
	Using toilet	3 (7.7)	1 (3.8)	
	Reaching for object	1 (2.6)	4 (15.4)	
	Sitting down or standing up	1 (2.6)	2 (7.7)	
	Using bedside commode	1 (2.6)	0 (0.0)	
	Unknown	19 (48.7)	13 (50.0)	
**Reason for activity**	Definitely elimination related	12 (30.8)	5 (19.2)	0.382
	Not elimination related	7 (17.9)	8 (30.8)	
	Unknown	20 (51.3)	13 (50.0)	
**Type of injury**	Fracture/dislocation	2 (5.1)	0 (0.0)	0.902
	Subdural hematoma and rib fracture	1 (2.6)	0 (0.0)	

Pain/swelling	5 (12.8)	2 (7.7)
Abrasion/skin tear	2 (5.1)	1 (3.8)
Bleeding	1 (2.6)	0 (0.0)
Contusion/hematoma	1 (2.6)	1 (3.8)
Nephrostomy catheter pulled out	1 (2.6)	0 (0.0)
Nothing	26 (66.7)	22 (84.6)

Table S2. Faller demographics

		Control group	Intervention group	p value
**Factor**	Number of cases	30	16	
**Age, median [range]**		82.50 [69.00–93.00]	86.00 [73.00, 98.00]	0.38
**Sex, n (%)**	Men	12 (40.0)	7 (43.8)	1
	Women	18 (60.0)	9 (56.2)	
**Ethnicity, n (%)**	Japanese	30 (100.0)	16 (100.0)	1
**Body mass index in kg/m^2, median [range]** *		18.97 [12.89–29.37]	20.17 [11.89, 28.94]	0.455
**FIM motor items score, median [range]** *		24.00 [13.00–57.00]	29.00 [13.00, 57.00]	0.435
**MMSE score, median [range]** *		16.00 [9.00–26.00]	22.00 [8.00, 26.00]	0.152
**Primary illness, n (%)**	Orthopedics or dermatology	7 (23.3)	4 (25.0)	0.953
	Various internal diseases **	17 (56.7)	10 (62.5)	
	Neurological disorder	4 (13.3)	1 (6.2)	
	Malignancy	2 (6.7)	1 (6.2)	
**Past history, n (%)**	Stroke	5 (16.7)	6 (37.5)	0.153
	Parkinson's disease	3 (10.0)	0 (0.0)	0.542
	Other neurological disease	4 (13.3)	2 (12.5)	1
	Diabetes mellitus	9 (30.0)	5 (31.2)	1
	Malignancy	2 (6.7)	2 (12.5)	0.602
**Medication within 7 days of hospitalization, n (%)**	Diuretics	8 (26.7)	7 (43.8)	0.325
	Antihypertensives except diuretics	11 (36.7)	5 (31.2)	0.757
	Anti-coagulant	5 (16.7)	7 (43.8)	0.077
	Diabetic medication	6 (20.0)	5 (31.2)	0.477
	BZD/Z-drugs	6 (20.0)	4 (25.0)	0.72
	Other hypnotics ***	9 (30.0)	9 (56.2)	0.116
	Antidepressants	1 (3.3)	1 (6.2)	1
	Anti-dementia medication	9 (30.0)	2 (12.5)	0.282
	Anticonvulsants	6 (20.0)	1 (6.2)	0.394
	Antipsychotics	11 (36.7)	4 (25.0)	0.52
	Anti-histamines	1 (3.3)	0 (0.0)	1
	Non-narcotic analgesics	0 (0.0)	2 (12.5)	0.116
	Narcotic analgesics	2 (6.7)	0 (0.0)	0.536
**Number of falls, median [range]**		1.00 [1.00–3.00]	1.00 [1.00–6.00]	0.928
**Length of stay, days**	Median [IQR]	23.50 [15.25–57.75]	40.00 [16.00–63.25]	0.426
	Mean (SD)	33.83 (24.54)	41.25 (29.62)	
**Outcome**	Discharge	19 (63.3)	9 (56.2)	0.274
	Transfer to a different ward	8 (26.7)	6 (37.5)	
	Transfer to a different hospital	3 (10.0)	0 (0.0)	
	Death	0 (0.0)	1 (6.2)	

[*] Excludes some missing data. BMI data were missing in 2 control and 2 intervention cases. FIM motor items score data were missing in one control and one intervention case. MMSE data were missing in nine control and five intervention cases.

[**] Includes cardiovascular, gastroenterology, otolaryngology, respiratory, and urology.

[***] Includes melatonin receptor agonists and orexin receptor antagonists.

FIM: Functional Independence Measure, MMSE: Mini-Mental State Examination, BZD: benzodiazepine, IQR: interquartile range, SD: standard deviation.
